# Increased rubidium levels in brain regions involved in food intake in obese rats

**DOI:** 10.1007/s00429-025-02930-8

**Published:** 2025-05-15

**Authors:** Magdalena Szczerbowska-Boruchowska, Aleksandra Chenczke, Blazej Ruszczycki, Pawel Wrobel, Wiktoria Tokarczyk, Patryk Stec, Katarzyna M. Sowa, Agata Ziomber-Lisiak

**Affiliations:** 1 Faculty of Physics and Applied Computer Science, AGH University of Krakow, Krakow, Poland; 2 SOLARIS National Synchrotron Radiation Centre, Jagiellonian University, Krakow, Poland; 3 Faculty of Medicine, Jagiellonian University Medical College, Krakow, Poland

**Keywords:** Brain structures, Rubidium, Appetite regulation, Brain activation, Obesity

## Abstract

The hypothalamus, particularly its ventromedial and lateral regions, plays a pivotal role in homeostatic appetite regulation and is therefore a significant brain structure in the development of obesity. Additionally, the development of obesity can be caused by improper hedonic regulation, which involves neural circuits and systems associated with pleasure and reward. Several studies indicate a possible link between rubidium (Rb) and obesity, despite this element is not being typically considered influential in vital life processes. The present study, therefore, aims to investigate whether excessive body fat in obese animals alters rubidium levels in brain regions directly or indirectly involved in appetite regulation. The research was conducted on high-calorie diet (HCD)-induced obese rats (OB, n = 8) and their lean counterparts (L, n = 8). The determination of Rb levels in brain areas was performed using synchrotron radiation-based X-ray fluorescence microanalysis (SRXRF). The obtained results show a significantly higher level of Rb in all brain areas examined, although the increase in this element in obese individuals was not the same in all structures. The largest relative difference (over 70%) was observed for the orbitofrontal cortex, and the smallest (about 35%) for the amygdala. Principal component analysis with linear projections demonstrated a clear differentiation between the brain structures of obese and non-obese individuals based on the full elemental composition of tissues, while Rb was the only element that distinguished the obese group in each of the examined brain structures. The results obtained clearly confirm the increase in Rb levels in the brain structures responsible for regulating appetite in obesity.

## Introduction

Appetite regulation is a multifaceted process governed by the interplay of physiological, hormonal, neural, and psychological mechanisms. It involves the coordination of several bodily systems that communicate to modulate hunger and satiety. Central to this process is the brain, particularly the hypothalamus, which serves as the primary control center for appetite regulation. The hypothalamus integrates input from various hormones, nutrients, and other brain regions to modulate food intake. The key regions within the hypothalamus, such as the ventromedial hypothalamus (VMH), often referred to as the satiety center, and the lateral hypothalamus (LH), known as the hunger center, play a pivotal role in the homeostatic regulation of appetite (Ahima and Antwi 2008). The goal of this homeostatic regulation is to maintain energy balance by ensuring the body consumes an adequate amount of food to meet its energy requirements, thereby preventing both undernutrition and overnutrition. This process is mediated by intricate interactions between hormones, the nervous system, and various physiological signals that work together to sustain a stable internal environment, particularly with respect to energy intake and expenditure (Schwartz et al. 2000).

While homeostatic regulation ensures that food intake meets energy requirements, hedonic regulation pertains to the influence of pleasure, reward, and emotional factors on eating behavior (Lutter and Nestler 2009). This process involves neural circuits and systems linked to pleasure and reward, shaping food preferences and consumption even in the absence of physiological hunger. The brain's reward system, particularly dopamine pathways, is crucial in the hedonic regulation of appetite. The mesolimbic dopamine system, which includes structures such as the ventral tegmental area (VTA), nucleus accumbens (NAc), and prefrontal cortex (PFC), is activated by rewarding stimuli like food. This system reinforces eating behavior, especially when food is perceived as pleasurable. The NAc plays a key role in reward processing, integrating signals from various brain regions to mediate the enjoyment of eating, particularly foods high in fats and sugars. It communicates with the PFC, which governs decision-making and cognitive control, affecting food choices based on expected rewards. Chronic hedonic eating, especially involving highly palatable foods, can result in addiction-like behaviors when the reward system becomes overstimulated and desensitized, requiring greater food consumption to achieve the same level of satisfaction. Over time, this can contribute to overeating and obesity (Avena et al. 2008).

Metabolic markers of obesity are indicators that reflect the body’s physiological response to excess fat accumulation and are used to assess the risk of obesity-related health issues such as type 2 diabetes, cardiovascular diseases, and metabolic syndrome. These markers typically involve blood tests or measurements that evaluate the body’s metabolic function, hormone levels, and organ activity. Common metabolic signs of obesity include elevated insulin, glucose, lipids (cholesterol, triglycerides), and inflammation (CRP) levels, all of which contribute to the risk of developing serious health problems associated with increased body fat. Experimental studies have shown (Szczerbowska-Boruchowska et al. 2023a) that a high-calorie diet (HCD)-induced obesity triggers structural changes related to lipids and proteins, as well as elemental redistribution in various brain regions crucial for energy balance. Specifically, alterations were observed in lipid unsaturation in the frontal cortex (FC) and VTA, changes in the fatty acyl chain length in the posterior lateral hypothalamus (PLH) and substantia nigra (SN), as well as modifications in the protein α-helix to β-sheet ratio and the percentage of β-turns and β-sheets in the NAc of obese animals. Notably, the levels of almost all measured brain biometals were reduced in obese animals, with phosphorus (P), potassium (K), and calcium (Ca) being the most distinguishing factors between lean and obese groups. Additionally, certain elements were altered in organs outside of the brain, including the liver, kidneys, adipose tissue, skeletal muscle, and heart (Ziomber-Lisiak et al. 2022a). Among the tested elements, rubidium (Rb) was the only one that showed an increase in the tissues of obese animals, despite a lower Rb intake in the diet. It is suspected that Rb may be a particularly important trace element in fetal development. In goats with Rb deficiency, growth retardation and an increased miscarriage rate have been observed (Zhao et al. 2024). A reduced level of Rb in the mother's amniotic fluid has also been found in newborns with low birth weight, as compared to a group of newborns with normal body weight. However, this situation has been reversed in the case of newborns with excessive birth weight (Ovadia et al. 2023).

Rb is a trace element found in the human body, with its content in adults estimated at approximately 0.36 ± 0.09 g (Lombeck et al. 1980). Elevation of Rb levels in the body may be caused by a diet rich in this element (Marsh 1986). Rb distribution in organs is similar to that of naturally occurring K, and Rb can substitute for K when deficiencies of the latter one occur (RELMAN 1956). Rb also has neurophysiological properties, influencing brain functions (Marsh 1986). The administration of rubidium to Macaca rhesus monkeys has been demonstrated to result in an elevation of behavioral activity and aggression, which has been accompanied by an increase in fluctuations in brain wave frequency as recorded by electroencephalograms (EEGs) (Meltzer et al. 1969). There is also evidence suggesting that Rb can replace potassium ions in the brain. It has been shown that the activity of the sodium–potassium pump (Na⁺/K⁺-ATPase) has increased by 35% in the presence of Rb, effectively displacing K in brain tissue samples. Additionally, the mere presence of Rb affects the increased activity of the sodium–potassium pump (Krulik et al. 1977). Thus, it is suspected that Rb transport occurs through the same mechanisms as in the case of K. Moreover, the similarity in the biochemical properties of K and Rb has been studied in the context of Alzheimer's disease (AD) diagnosis (Roberts et al. 2016). It has been found that Rb and K levels are reduced in the frontal cortex of AD patients. ROC curve analysis (Receiver Operating Characteristic Curve) identified Rb as a better marker for predicting Alzheimer's disease. A strong positive correlation between K and Rb was also demonstrated in both the control and diseased groups. It was suggested that a decrease in Rb levels could serve as a marker for AD, as its interchangeability with K ions effectively reflects sodium–potassium pump dysfunction.

It has been shown that Rb levels in the body also increase during the occurrence of oxidative stress, which damages DNA and tissues. G. Barrientos et al. demonstrated that lipid peroxidation products, as well as changes in antioxidant substances, are associated with the amount of this element (Barrientos et al. 2020). In endurance athletes, a positive correlation was found between 3,4-methylenedioxyamphetamine (MDA) and Rb levels. MDA is a product of lipid peroxidation, leading to the formation of reactive oxygen species. A positive relationship between the levels of Rb and α-tocopherol, an antioxidant, was also observed. Thus, the increase in Rb levels is explained by stimulation of sodium–potassium pump in response to free radicals (Barrientos et al. 2020).

Motivated by the findings of our previous research on changes in Rb levels in obesity, as well as the established role of Rb in neurophysiological processes and brain activity as shown by other studies, the aim of the present paper is to investigate whether Rb levels in brain regions directly or indirectly involved in appetite regulation are altered by excessive body fat in obese animals. To achieve the stated goals, synchrotron radiation-based X-ray fluorescence (SRXRF) was employed. The SRXRF technique was previously satisfactorily applied to elemental studies of thin tissue sections (Kastyak et al. 2010; Ziomber et al. 2018; Kasprzyk et al. 2022). This research technique, due to its low detection limits for trace elements as well as its sufficient spatial resolution at microscopic level, is a unique analytical tool also for the determination of Rb levels in rodent brain structures.

## Methods

### Sample obtaining and preparation

The animal brain tissue for the SRXRF analysis was obtained from 16 male Wistar rats, divided into two groups: the lean group (L, n = 8) and the obese group (OB, n = 8). They were the same animals described in our previous work (Szczerbowska-Boruchowska et al. 2023b). Obesity was induced over a period of 54 days using a high-calorie diet that contained nearly three times more fat, as compared to the standard diet (Ziomber-Lisiak et al. 2022b). Lean and obese subjects exhibited significant differences in final body weight, feed intake, and epididymal fat pad weight. Further details can be found elsewhere (Szczerbowska-Boruchowska et al. 2023b). After euthanizing the animals, the brains were quickly removed, frozen, and stored for further analysis. 

**Fig. 1:**
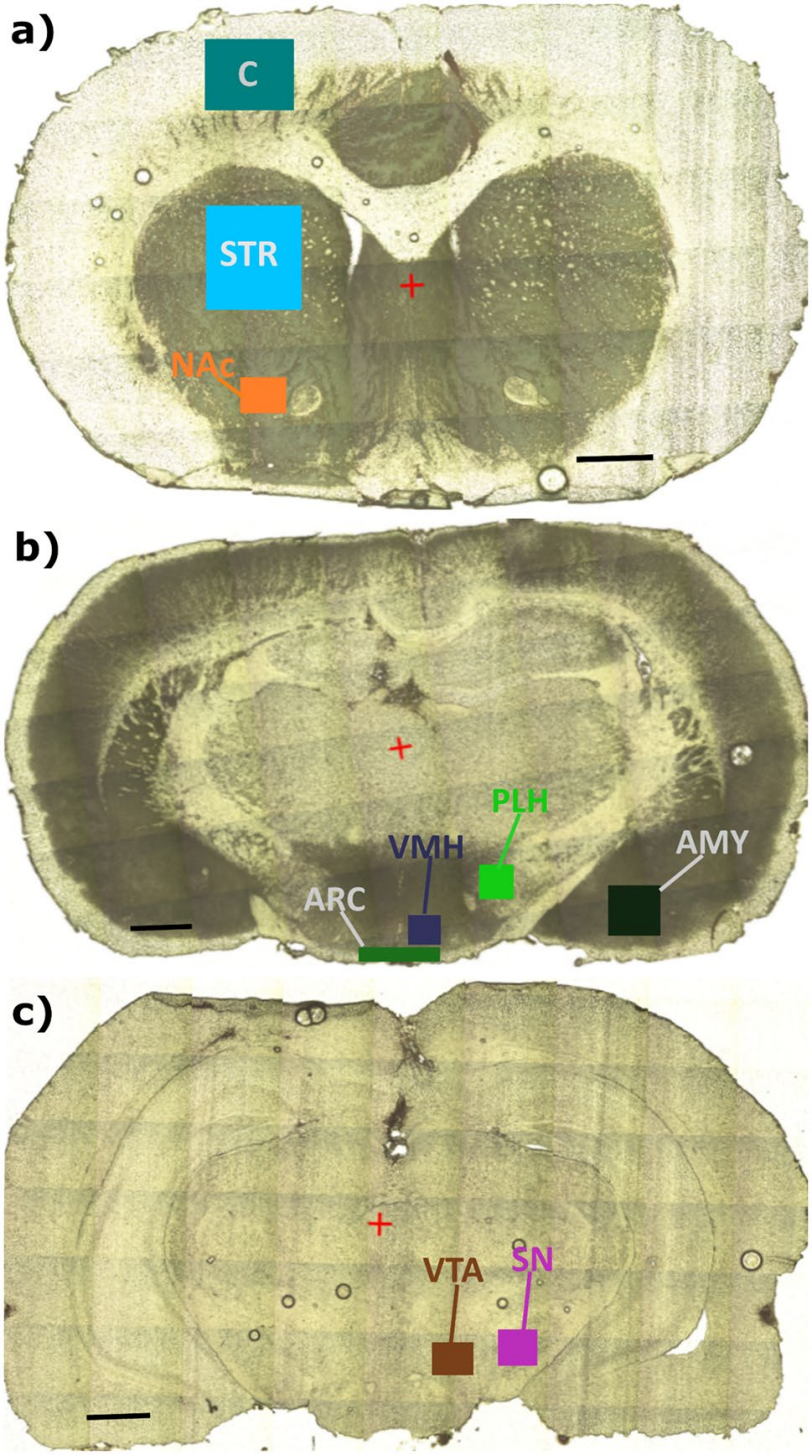
Microscopic images of brain slices with marked regions responsible for appetite regulation, which were analyzed using SRXRF: **a** *C* orbitofrontal cortex, *STR* striatum, *NAc* nucleus accumbens; **b** *VMH* ventromedial hypothalamus, *ARC* arcuate nucleus of the hypothalamus, *PLH* posterior lateral hypothalamus, *AMY* amygdala; **c** *VTA* ventral tegmental area, *SN* substantia nigra. Elaborated basing on (Paxinos and Franklin 2019)

The investigation focused on specific brain regions that are directly and indirectly involved in appetite regulation, including the amygdala (AMY), the ventromedial hypothalamus (VMH, satiety center), the posterior lateral hypothalamus (PLH, hunger center), the arcuate nucleus of the hypothalamus (ARC), the striatum (STR), the nucleus accumbens (NAc), the orbitofrontal cortex (C), the ventral tegmental area (VTA), and the substantia nigra (SN). Three thin slices of brain tissue containing the above-mentioned brain structures were used for each rat in the study. For this purpose the specimens were cryo-sectioned at − 18 °C onto 20 µm—thick slices and mounted into Ultralene membranes (SPEX SampePrep). Microscopic images of brain sections used in the study are presented in Fig. 1. The experiment was conducted in accordance with the National Guide for the Care and Use of Laboratory Animals, and was approved by the Local Ethical Committee on Animal Testing at the Jagiellonian University in Krakow, Poland (approval no. 157/2013).

### SRXRF measurements

The experiment was performed at the POLYX beamline (Sowa et al. 2023) at SOLARIS National Synchrotron Radiation Centre (Szlachetko et al. 2023) in Krakow. A Monochromatic beam of 15.4 keV energy from SOLARIS bending magnet (1.3 T) was acquired with Mo/B_4_C multilayer monochromator with 1.3% bandwidth. The beam was focused with use of polycapillary optics (Helmut Fischer GMBH, 114 msl01-1, 40 mm working distance) to achieve the spot size of the diameter around 100 µm (FWHM). Tissue samples were maintained in the atmospheric air with a 45/45 deg geometry. The Hitachi Vortex EM360 silicon drift detector (ML3.3 extreme window, 0.5 mm thick active area 100 mm^2^) coupled to XGLab Dante digital pulse processor was used to record the spectral response from the sample. For each brain area, four point measurements have been performed (typically on 100 × 100 µm^2^ or 500 × 500 µm^2^ mesh, depending on the brain structure), separately for left and right hemispheres. In addition, to obtain surface masses of Rb as well as other tissue elements measured simultaneously, a set of Micromatter XRF Calibration Standards with elements deposited on thin Mylar film was measured. For each standard mesh, 9 points were measured. The time of XRF spectrum acquisition was around 120 s and 40 s per single point for tissues and calibration standards, respectively.

### Spectral analysis and data treatment

**Fig. 2:**
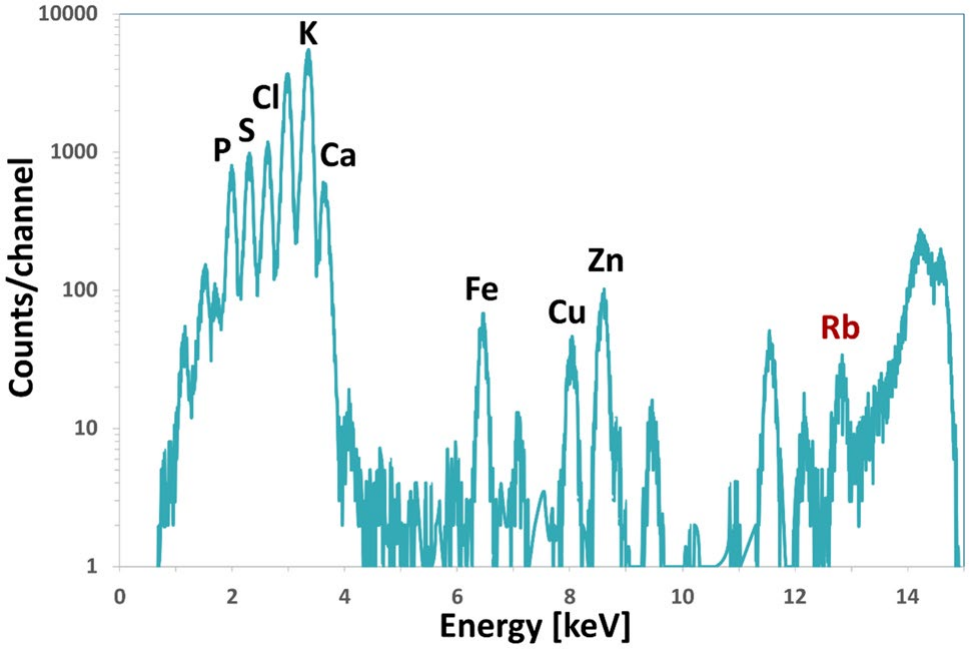
Exemplary summation spectrum recorded for rat brain tissue using the SRXRF method. The Kα lines of the identified elements: P, S, Cl, K, Ca, Fe, Cu, Zn, and Rb were marked

After the measurements, registered spectra (c.f. Fig. 2) were normalized to the incident X-ray beam flux and detector live-time. Subsequently, the curve fitting and batch–fitting procedures performed using the PyMca software (Solé et al. 2007) were used for extracting the net areas of K_α_ lines of elements. Elemental mass deposits per unit area were determined using external standard procedure, as described elsewhere (Szczerbowska-Boruchowska 2012; Surowka et al. 2018). Taking into account the tissue thickness, the calculations according to the thin sample approach were performed. Detection limit (LOD) of Rb was calculated according to the formula given by Currie (Currie 1968). Hereby, LOD Rb was calculated as the average value of 10 randomly chosen spectra.

### Statistical procedures

For each brain region within a given study group, surface mass values of Rb and other elements were determined based on the summation spectra. The resulting data, characterizing each region for individual subjects within the studied animal group, were subjected to statistical analysis.

Results of intergroup comparisons are presented as the mean (± standard deviation). To assess the significance of differences in Rb levels in the brain structures of individuals from groups L and OB, a Student's t-test was used. To evaluate the influence of factors such as “group”, “structure”, and “brain hemisphere” on Rb levels, a nonparametric equivalent of analysis of variance (Aligned Rank Transform tool (ARTool)) (Wobbrock et al. 2011) was applied, due to the lack of fulfillment of the assumption of equal variances for all data used in the analysis. Outlier rejection was performed using a normal two-sided test. The data distribution conformity was verified using the Shapiro–Wilk test (W-S). The assessment of differences in Rb levels between brain structures within a given animal group was conducted using multivariate analysis of variance (ANOVA) with Tukey’s post-hoc tests, provided that the assumptions of normal distribution and equal variances were met (for group L). Data that did not meet the assumption of equal variances (group OB) were analyzed using the nonparametric Kruskal–Wallis test (K-W). Since the SRXRF technique is a multi-element technique, multivariate data exploration methods were applied based on the information provided by the SRXRF spectrum on the levels of other elements measured alongside Rb. These methods included principal component analysis (PCA) complemented by linear projections. The analysis was conducted (1) to determine whether, based on the complete elemental composition measured in different brain regions, a separation of the studied groups can be observed, and thus whether it can be concluded that the elemental composition of the tissues is specific/unique to a given group of animals; (2) to assess the significance of Rb in comparison to other elements in the elemental characterization of the OB group (whether Rb can be considered an attribute of a given group of obese individuals, in other words). In all statistical tests, we adopted a significance level of 0.05. S-W tests, Student's t-test, ANOVA, and K-W tests were performed using Statistica v. ver. 13.3 (Tibco Software Inc., Statsoft, Poland). The analysis using the ARTool tool was conducted using custom written software in R 4.2.2. PCA analysis and linear projections were performed using Quasar v. 1.10.1. (Toplak et al. 2021).

## Results

A typical SRXRF spectrum obtained for rat brain tissue is presented in Fig. 2.

Under the applied measurement conditions, it was possible to detect the following elements: P, S, Cl, K, Ca, Fe, Cu, Zn, and Rb. Additionally, Mn and Br were also detected in some samples. However, since these elements were not present in all analyzed samples, they were not included in the PCA analysis.

The analysis using ARTool demonstrated a statistically significant effect of the group factor (p < < 0.05) and the region factor (p = 0.0032) on Rb levels. No significant effect of the brain hemisphere on the measured element was found (p = 0.49). Additionally, no significant interaction between the analyzed factors on Rb levels was observed.

**Fig. 3:**
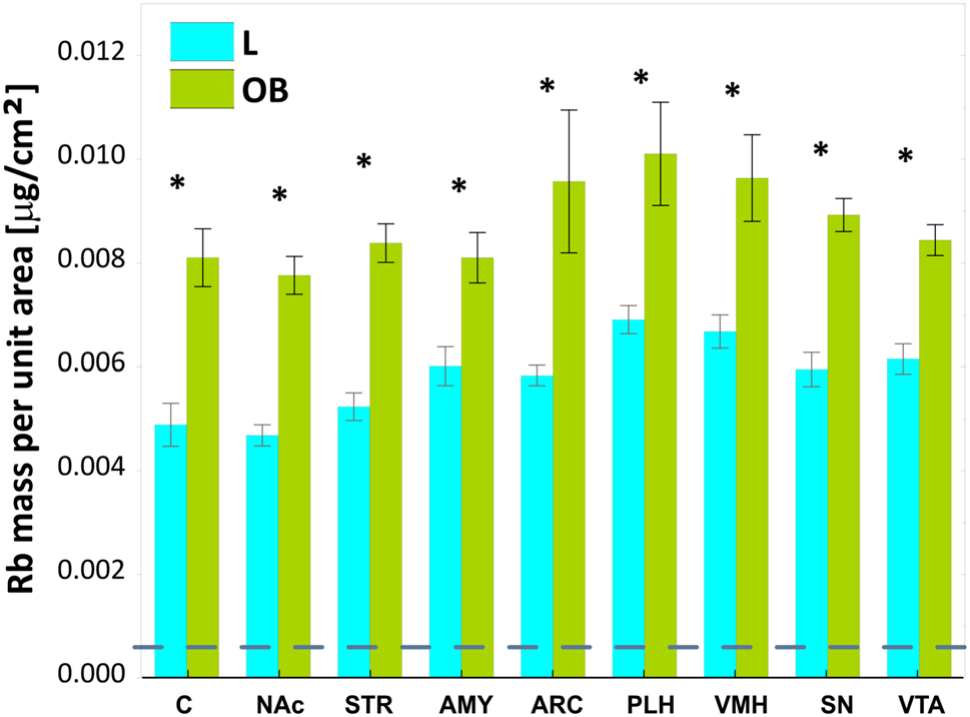
Comparison of the average surface masses of rubidium in brain areas directly and indirectly responsible for appetite regulation between the L and OB groups. *L* group control, *OB* obese group, *C* orbitofrontal cortex, *NAc* nucleus accumbens, *STR* striatum, *AMY* amygdala, *ARC* arcuate nucleus of the hypothalamus, *PLH* posterior lateral hypothalamus, hunger center, *VMH* ventromedial hypothalamus, satiety center, *SN* substantia nigra, *VTA* ventral tegmental area. Whiskers: range mean ± standard error; *statistically significant results based on Student's t-test (p < 0.05). The dotted line shows the detection limit (LOD) of rubidium

As a result, further statistical analysis was conducted without distinguishing between the right and left brain hemispheres. The average surface mass values of Rb between groups L and OB are presented in Fig. 3.

All surface mass values of Rb under the applied measurement conditions significantly exceeded the detection limit of this element, which was 0.000623 ± 0.000049 μg/cm^2^ (c.f. Fig. 3).

The results of surface mass measurements for the remaining elements can be found in study (Szczerbowska-Boruchowska et al. 2023a). The significance of differences in Rb levels across the studied brain regions between groups L and OB, based on Student’s t-test, is indicated in Fig. 3. The obtained results show significantly higher Rb levels in all brain structures involved in appetite control in individuals on an HCD diet compared to those on a standard diet; AMY p = 0.0015; ARC p = 0.0056; C p < 0.001; NAc p = < 0.001; PLH p = 0.0027; SN p = < 0.001; STR p = < 0.001; VMH p < 0.001; VTA p = < 0.001).

**Fig. 4:**
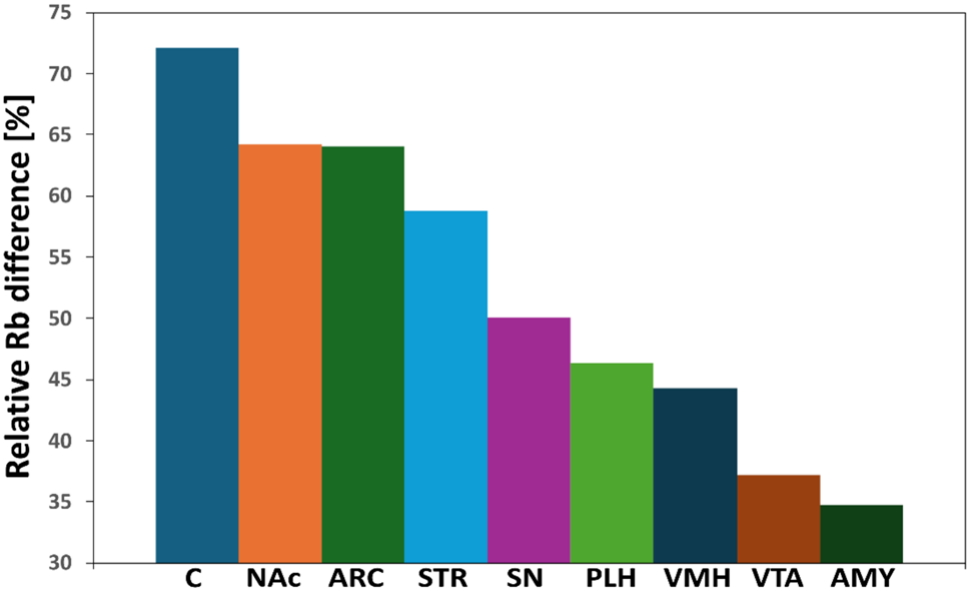
Relative percentage differences in the average surface masses of rubidium between the OB (obese) group and the L (control) group in brain areas responsible for appetite regulation. *C* orbitofrontal cortex, *NAc* nucleus accumbens, *STR* striatum, *AMY* amygdala, *ARC* arcuate nucleus of the hypothalamus, *PLH* posterior lateral hypothalamus, *VMH* ventromedial hypothalamus, *SN* substantia nigra, *VTA* ventral tegmental area

Figure 3 also indicates that in the group of non-obese individuals, the hunger and satiety centers exhibit the highest Rb levels, compared to the other structures. Conversely, the lowest Rb level in this experimental group was observed in STR. In the group of obese individuals, the Rb levels were similarly highest in the PLH and VMH regions. However, there were no significant differences relative to the other regions (see the results below). Figure 4 shows the relative percentage differences in the average surface masses of Rb between the OB and L groups, for each structure. The largest relative difference (approximately 72%) was observed in the region C, whereas the smallest relative difference (approximately 35%) was observed in the AMY. The relative differences in Rb levels between the OB and L groups for the hunger center (PLH) and the satiety center (VMH) were approximately 46% and 44%, respectively.

One-way ANOVA showed statistically significant differences (F = 5.25, p < 0.001) in Rb levels between the analyzed brain regions in the control group. The Tukey post-hoc test for multiple comparisons revealed that Rb levels differed significantly between C and PLH (p < 0.001), C and VMH (p < 0.001), NAc and PLH (p = 0.0025), NAc and VMH (p = 0.011), and STR and PLH (p = 0.013).

In contrast, the Kruskal–Wallis rank ANOVA for the obese group did not show significant differences in surface mass values of Rb between different brain regions in obese individuals (H = 11.0, p = 0.20).

**Fig. 5:**
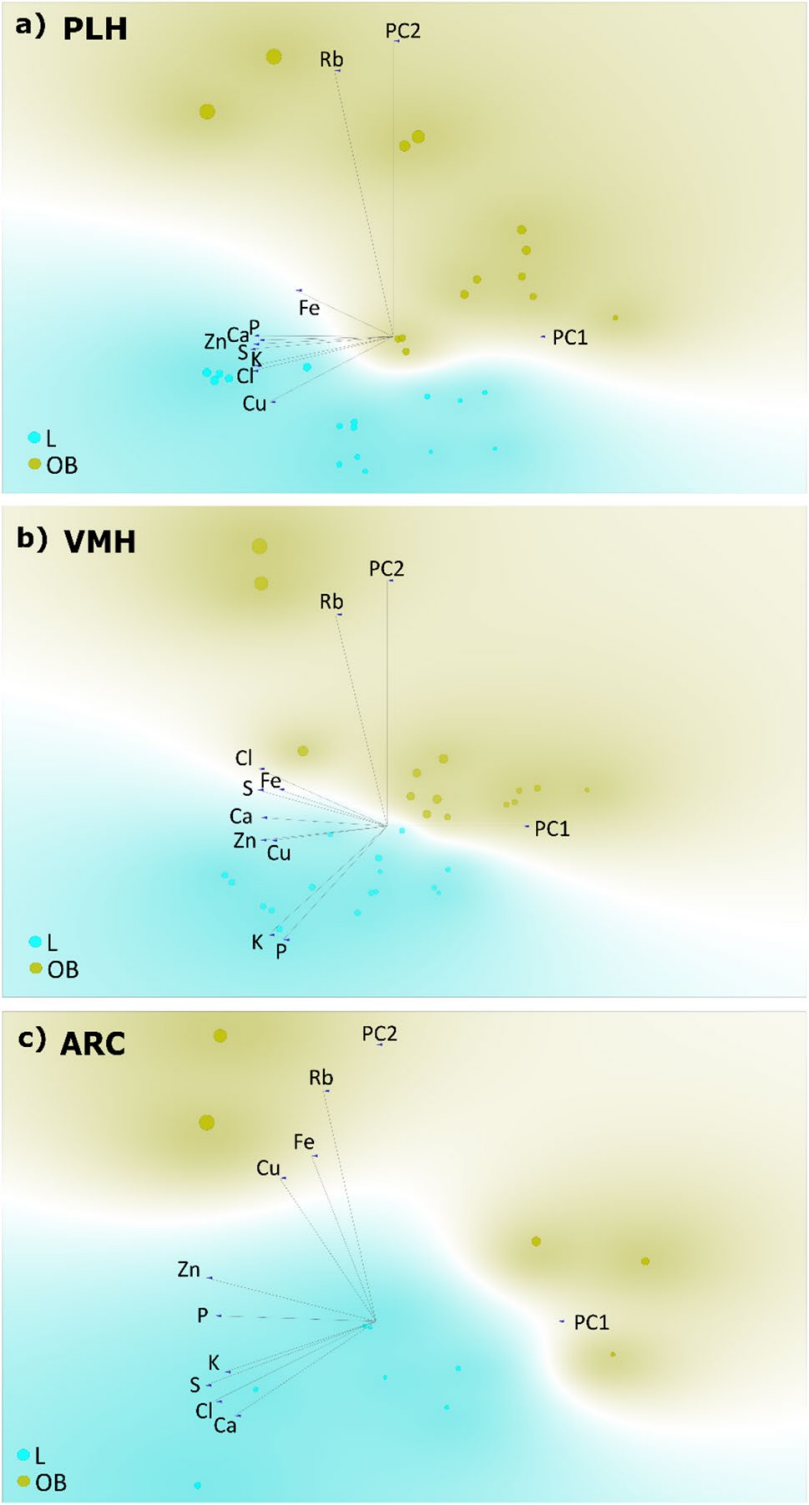
Results of principal component analysis (PCA) performed based on the average surface masses of elements determined in brain areas directly associated with appetite regulation: **a** *PLH* posterior lateral hypothalamus; **b** *VMH* ventromedial hypothalamus; **c** *ARC* arcuate nucleus of the hypothalamus. *L* control group, *OB* obese group

**Fig. 6:**
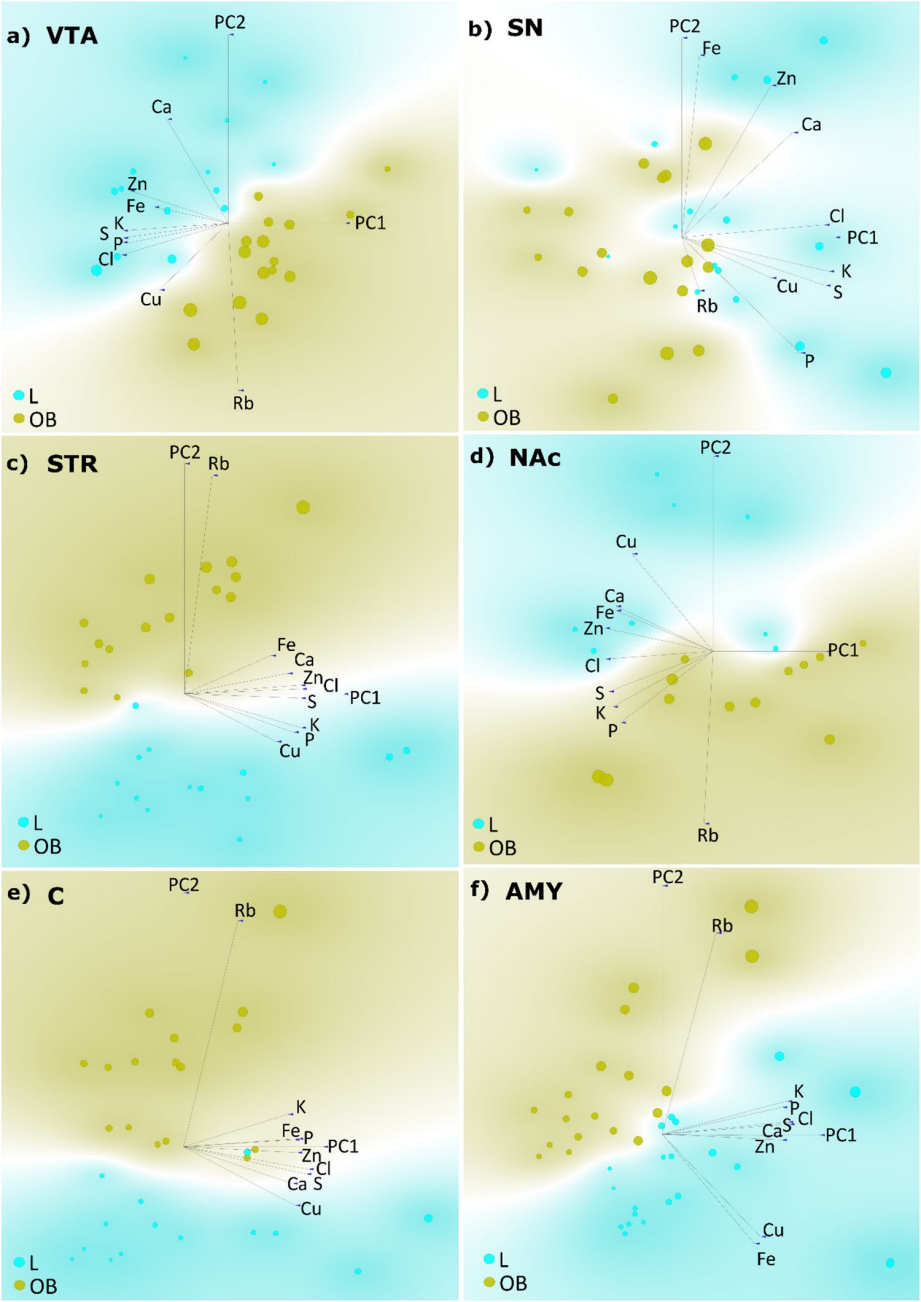
Results of principal component analysis (PCA) performed based on the average surface masses of the identified elements in brain areas indirectly associated with appetite regulation. **a** *VTA* ventral tegmental area, **b** *SN* substantia nigra, **c** *STR* striatum, **d** *NAc* nucleus accumbens, **e** *C* orbitofrontal cortex, **f** *AMY* amygdala. *L* control group, *OB* obese group

Using the complete elemental composition of the studied brain regions, the differentiation of tissues from individuals in groups L and OB was assessed. The significance of Rb relative to other elements in the characterization of brain regions from obese individuals was verified using linear projections. The results are presented in Figs. 5 and 6 for structures directly and indirectly related to appetite regulation, respectively. Additionally the summary of the results of the PCA complemented by linear projections are summarized in Table 1. In all structures directly related to appetite regulation (the PLH, ARC, and VMH regions), a clear distinction between the groups can be observed. The variances of the data are respectively being explained by the principal components PC1 and PC2 (Fig. 5) as 87%, 88%, and 76%. The analysis shows that in the hunger center (Fig. 5a) and in the satiety center (Fig. 5b), Rb is the element most strongly associated with the OB group, when considered in comparison to other elements. In the ARC (Fig. 5c), Rb is also strongly linked to the OB group. It is also worth noting its strong correlation with Cu and Fe, however no strong correlations between Rb and the other elements were observed in the studied brain structures.

**Table 1:** Summary of the results of the principal component analysis (PCA) complemented by linear projections

Brain structure	Group-indicating element		PCA group separation
	L	OB	
PLH	P, Ca, Zn, S, K, Cl, Cu	Rb	Yes
VMH	Ca, Zn, Cu, K, P, Cl, S	Rb	Yes
ARC	Zn, P, K, S, Cl, Ca	Rb, Cu, Fe	Yes
VTA	Ca, Zn, Fe, K, S, P, Cl	Rb	Yes
SN	Zn, Ca, Cl, K, S, Cu, P	Rb	Yes
STR	Cu, P, K	Rb, Fe, Ca	Yes
NAc	Cu, Ca, Fe, Zn, Cl	Rb, S, K, P,	Yes
C	Cu, Ca	Rb, K	Yes
AMY	Fe, Cu, Zn, Ca, S, Cl, P, K	Rb	Yes

*PLH* posterior lateral hypothalamus, *VMH* ventromedial hypothalamus, *ARC* arcuate nucleus of the hypothalamus, *VTA* ventral tegmental area, *SN* substantia nigra, *STR* striatum, *NAc* nucleus accumbens, *C* orbitofrontal cortex, *AMY* amygdala, *L* control group, *OB* obese group

Figure 6 shows PCA results for the regions indirectly involved in hunger and satiety control. For the VTA (Fig. 6a) the PCA shows a clear separation between the studied groups, with the variance explained by PC1 and PC2 totaling 69%. Regarding the VTA, Rb is the only element distinguishing the OB group and describes it very well, as indicated by the length of the loading vector. It can also be noted that there are rather strong negative correlations between Rb and both Ca and Fe. In the case of SN (Fig. 6b), a distinction between the groups emerges, with the primary components explaining a total of 71% of the variance. Similarly, Rb exhibits the highest affinity for distinguishing the obese group. However, the length of the Rb vector suggests that the influence of Rb in differentiating the groups is not as strong as in the case of the other structures. In the case of SN, a strong negative correlation between Rb and Cl can be observed. Based on the projection for STR (Fig. 6c), both principal components explain a total of 74% of the variance of the results. In this case, a very clear distinction between the control and obese groups can be observed, as described by principal component PC1. Similarly, Rb relative to the other elements, mostly indicates the OB group. For the NAc (Fig. 6d) 86% of the data variability was explained by two principal components, PC1 and PC2. The separation between the L and OB groups is easily noticeable, as is the significance of Rb in defining the OB group. Apart from its negative correlation with Cu and Ca, no other stronger association of this element with the remaining ones can be identified. The projection for the C (Fig. 6e), has a similar character to that for the NAc (cf. Figure 6d), with a similar level of data variability – 83% explained by PC1 and PC2. Once again, the boundary between the groups was quite well marked, and a strong association of Rb presence with the OB group was observed. At the same time, no evident correlations of Rb with P, Cu, S, K, Cl, Ca, Zn, or Fe were found. Figure 6f presents the projection made for AMY. It is characterized by 80% of the variability being explained by PC1 and PC2. Rb does not show any closer correlation with the other measured elements; however, against their background, it best defines the obese group, which is clearly separated from the control group.

## Discussion

The studies aimed at assessing the amount of Rb in specific brain structures were expected to determine the role of this element in the early stage of obesity and to quantify how well it characterizes the obese group.

Numerous studies have indicated a link between obesity and impaired brain function (Szczerbowska-Boruchowska et al. 2023a), (Barakat et al. 2024), (Loper et al. 2021), as well as the occurrence of neurodegenerative diseases (Neto et al. 2023), (Kueck et al. 2024). Since Rb itself has been increasingly studied in the context of its role in the organism, it has been suggested as a potential marker of obesity (Szczerbowska-Boruchowska et al. 2023a),(Szczerbowska-Boruchowska et al. 2023b), (Tinkov et al. 2015), (Ziomber-Lisiak et al. 2022a), (Amerikanou et al. 2023). Essentially, it has been shown that at the early stage of obesity development, there is a significant increase in Rb levels across all examined brain structures (cf. Figure 3). It could have been speculated that the observed significant increase in Rb could be diet-induced, as its absorption through intestinal villi into the bloodstream might lead to elevated levels in the organism (Lombeck et al. 1980), (Shao et al. 2017), (Tinkov et al. 2015). However, this hypothesis can be rejected, as our previous study (Ziomber-Lisiak et al. 2022a) using the same methodology demonstrated that Rb intake from food was lower in OB group subjects than in the control group. Therefore, it can be suggested that the accumulation of Rb in tissues is a mechanism in response to a HCD and obesity. Since it has been shown that Rb, under certain circumstances, replaces K in the organism, which is essential for the proper functioning of the sodium-K pump (RELMAN 1956), (Krulik et al. 1977) and obesity is characterized by K deficiency in the body (Szczerbowska-Boruchowska et al. 2023a), the changes in Rb levels in tissues of obese individuals may reflect impaired Na⁺/K⁺-ATPase activity, leading, among other things, to the formation of reactive oxygen species (ROS) in the organism (Amin et al. 2020). Indeed, a positive correlation has been demonstrated between Rb levels and the oxidative stress marker MDA in athletes (Barrientos et al. 2020), suggesting that under extreme conditions, Rb plays a role in supporting the function of Na⁺/K⁺-ATPase. Thus, analyzing our results, it can be speculated that the increased values of mean surface masses of Rb in the examined brain structures of obese individuals may result, at least in part, from a response to oxidative stress, initiated by the accumulation of excess fat tissue. Although the PCA analysis did not reveal a strong correlation between Rb and K, it did confirm that in the areas of PLH, VMH, ARC, STR, AMY, SN, and VTA, the control group exhibited higher levels of K compared to the obese group (cf. Figs. 5 and 6). Indeed, significantly lower levels of K in PLH, VMH, AMY, VTA, and SN in obese individuals fed a HCD were presented in our previous work (Szczerbowska-Boruchowska et al. 2023a). It is possible that at a more advanced stage of obesity, the aforementioned correlations could have been confirmed.

Furthermore, attention should be drawn to the neurophysiological properties of Rb (Marsh 1986), (Zhao et al. 2024), (Hao et al. 2022). Changes in its mean surface mass values for brain areas responsible for regulating hunger and satiety in the obese group may affect the disrupted communication between them.

Hypothalamic structures such as PLH and VMH are sensitive to the systematic delivery of saturated fats from the diet, which can indirectly lead to inflammation and neuronal damage (de Mello et al. 2018). The frontal cerebral cortex generally shows lower activity in response to a high-calorie diet (Val-Laillet et al. 2011), but it has been shown that in obese individuals, its activity increases after a meal. This is explained by the fact that the cerebral cortex, responsible for inhibiting impulsive decisions such as overeating, exerts increased activity to suppress the activity of other areas responsible for appetite regulation. Additionally, it has been hypothesized that the C is an important regulator in the termination of the eating process, as it acts antagonistically on orexigenic neurons located in the thalamus, hypothalamus, and basal ganglia (Del Parigi et al. 2002). Basing on this theory, and by analyzing the results of comparisons of Rb levels in brain structures within the given group, it can be observed that the natural difference in the amount of Rb between the C and PLH and VMH has been lost, which may be a signal confirming disrupted communication between these areas, resulting in insufficient inhibition of eating and the development of obesity. It should be also noted that for the C, the greatest relative difference in Rb levels was observed between the group of lean and obese individuals (Fig. 4). Increased values of mean surface masses of Rb for the OB group were also observed in the areas of the reward system and mesolimbic pathways (AMY, VTA, SN, NAc, and STR), which play an important role in dopamine (DA) processing (Wang et al. 2001) and also include cortical areas (Volkow et al. 2011). Parts of the hypothalamus and the ARC communicate with these areas primarily through neuropeptides that regulate food intake, among other signaling pathways (Wallace and Fordahl 2022). In obesity, a decrease in the availability of dopaminergic (D2) receptors has been observed, which is a characteristic feature of addiction states. DA supplied through food enhances feelings of satisfaction and pleasure while simultaneously leading to the dysregulation of these receptors, primarily located in the SN and VTA, due to their excessive stimulation (Szczerbowska-Boruchowska et al. 2023a), (Wang et al. 2001). Over time, this leads to a state of"adaptation"where the released DA no longer produces the same level of euphoria or satisfaction. At this stage, the organism responds not only to the taste of food but also to its sight, smell, or even the memory of it, with dopaminergic connections in the AMY and NAc playing a key role (Volkow et al. 2011).

It should be also noted that the Tukey test results for NAc and STR in the control group revealed significant differences in Rb levels with brain regions directly involved in appetite regulation. Similarly to the C, these differences disappeared in the obese group, and the Rb levels in these structures were relatively high compared to the control group (Fig. 4). These results could suggest that, at the early stage of obesity, Rb homeostasis is disrupted in reward-related areas and in the mesolimbic pathways, which could promote obesity. Given that Rb may potentiate the effects of D1 and D2 receptor antagonists (Dehpour et al. 1998), it cannot be ruled out that it may also influence the bioavailability of these receptors in the brain. Elevated mean surface masses of Rb in the aforementioned brain regions observed in the OB group could have indicated reduced bioavailability of receptors for DA, as well as disrupted communication between dopaminergic and reward-related areas (hedonic regulation) areas and the structures responsible for the homeostatic regulation of appetite (PLH, VMH, ARC).

It should be pointed out that, as the influence of the estrous cycle on the level or metabolism of Rb in the brain has not been studied in our research, we limited ourselves to male subjects. Although estrogen is recognized for its protective role in the nervous system as well as its effects on brain growth and function, there is currently no scientific evidence establishing a direct connection between estrogen and rubidium levels or its metabolism in the brain. Estrogen, especially in the form of estradiol, regulates nerve cell activity, supports the formation of new neurons, and plays a role in cognitive functions (Bustamante-Barrientos et al. 2021). Various forms of modulation, such as neuronal firing patterns, synaptic plasticity, and structural changes, have been shown to vary in relation to the stages of the estrous cycle (Inoue 2022). In addition, both estrogen and progesterone can affect neurogenesis and neurotrophic gene expression in the hippocampus (Scharfman et al. 2003). It was also shown that estrogen signaling can influence the excitability of dopamine neurons in the VTA, impacting stress and reward processing (Scharfman et al. 2003) and therefore potentially feeding behavior (Ma et al. 2020). However, no research to date indicates a direct relationship between estrogen and rubidium, nor does it suggest that estrogen influences the distribution or metabolism of rubidium in the brain. Furthermore, we based our research on male subjects only, taking into account our previous observations (Ziomber-Lisiak et al. 2022b). The experimental results have proven that male Wistar subjects gain weight faster compared to female ones, so they are a better model of obesity than females.

## Conclusions

The present study shows that HCD-induced obesity is accompanied by changes in Rb levels in the brain tissue, affecting structures involved in both homeostatic and hedonic appetite regulation. In particular, significantly elevated levels of Rb were found in all the examined structures, i.e., C, NAc, STR, PLH, VTA, ARC, AMY, SN, and VTA, in obese individuals compared to their non-obese counterparts.

It should be noted that the increase in Rb levels was not uniform across all structures. The greatest relative difference in Rb levels between individuals in groups OB and L was observed in the C (approximately 72%), while the smallest difference was found in the amygdala (approximately 35%). Changes in the hunger center (PLH) and satiety center (VMH) were at the level of 45%.

Additionally, the performed PCA analysis based on the levels of all elements measured using the SRXRF technique in the examined tissues (P, S, Cl, K, Ca, Fe, Cu, and Zn) confirmed that Rb is the element that best indicates obesity, compared to other elements. It also showed that complete elemental composition of the tissues differs and allows for a clear distinction between individuals from groups L and OB. The mechanisms leading to an increase in Rb amount in brain regions responsible for appetite regulation in response to a HCD are still unknown, however, it cannot be ruled out that the neurophysiological properties of Rb and its similarity to K ions may influence communication between the studied brain structures. The obtained results are consistent with the findings of our previous studies, where we demonstrated an increase in Rb levels in most tissues and organs of obese individuals, indicating Rb as a marker of metabolic disturbances induced by excessive fat tissue. The present study confirms a similar relationship at the level of brain tissue.

We believe that the obtained results provide a valuable contribution to understanding the mechanisms responsible for regulating the body's energy balance and the pathogenesis of obesity, which is essential for preventing obesity development and its negative consequences.

## Data Availability

The data that support the findings of this study are available from the corresponding author upon reasonable request.
